# Improving eSource Site Start-Up Practices

**DOI:** 10.21203/rs.3.rs-4414917/v1

**Published:** 2024-05-24

**Authors:** Amy E. Cramer, Linda S. King, Michael T. Buckley, Peter Casteleyn, Cory Ennis, Muayad Hamidi, Gonçalo M. C. Rodrigues, Denise C. Snyder, Aruna Vattikola, Eric L. Eisenstein

**Affiliations:** Johnson & Johnson Innovative Medicine; Astellas Pharma; Memorial Sloan Kettering Cancer Center; Johnson & Johnson Innovative Medicine; Duke University School of Medicine, Vice Dean’s Office; UT Health San Antonio; Janssen-Cilag, S.A; Duke University School of Medicine, Duke Office of Clinical Research; Merck & Co., Inc; Duke University

**Keywords:** clinical research, clinical trial, electronic data capture, electronic health records, health information technology, eSource

## Abstract

**Background:**

eSource software that copies patient electronic health record data into a clinical trial electronic case report form holds promise for increasing data quality while reducing data collection, monitoring and source document verification costs. Integrating eSource into multicenter clinical trial start-up procedures could facilitate the use of eSource technologies in clinical trials.

**Methods:**

We conducted a qualitative integrative analysis to identify eSource site start-up key steps, challenges that might occur in executing those steps, and potential solutions to those challenges. We then conducted a value analysis to determine the challenges and solutions with the greatest impacts for eSource implementation teams.

**Results:**

There were 16 workshop participants: 10 pharmaceutical sponsor, 3 academic site, and 1 eSource vendor representatives. Participants identified 36 Site Start-Up Key Steps, 11 Site Start-Up Challenges, and 14 Site Start-Up Solutions for eSource-enabled studies. Participants also identified 77 potential impacts of the Challenges upon the Site Start-Up Key Steps and 70 ways in which the Solutions might impact Site Start-Up Challenges. The most important Challenges were: (1) not being able to identify a site eSource champion and (2) not agreeing on an eSource approach. The most important Solutions were: (1) vendors accepting electronic data in the FHIR standard, (2) creating standard content for eSource-related legal documents, and (3) creating a common eSource site readiness checklist.

**Conclusions:**

Site start-up for eSource-enabled multi-center clinical trials is a complex socio-technical problem. This study’s Start-Up Solutions provide a basic infrastructure for scalable eSource implementation.

## INTRODUCTION

Clinical trial productivity continues to decline while complexity and costs are escalating (1–4).

Site-based activities account for a disproportionate share of total clinical trial costs and frequently are targets of cost-reduction efforts (5–6). eSource software that copies patient electronic health record data into a clinical trial electronic case report form holds promise for increasing data quality while reducing data collection, monitoring and source document verification costs (7–8). However, few sites are prepared to use eSource in multicenter studies (9). Integrating eSource into clinical trial start-up procedures could provide a means to prepare sites for participation in eSource clinical trials.

Clinical trial site start-up is a difficult and time-consuming process that may be associated with significant time delays (10). Site activation time is inversely related to enrollment rates and is seen as a key driver of clinical trial success (11–12). A recent Clinical Trials Transformation Initiative (CTTI) study found that the median time from site protocol receipt to first patient enrollment was 128 days (IQR, 0–1192 days) (13). Of this time, the greatest time interval was between contract execution and first patient enrollment (median, 87 days; IQR, 0–2928 days). Factors associated with start-up delays in this study included: regulatory, contracts and budgets, insurance, clinical supplies, site identification and selection, site activation, and inefficient processes/pitfalls (14). Many of these factors also may be impacted by eSource implementation. Although previous research has identified best practices for accelerating clinical trial set-up and conduct, none have investigated the integration of eSource with traditional clinical trial start-up procedures (15). We conducted a study to determine how eSource site start-up practices could be improved. We began by identifying eSource site start-up key steps, major challenges that occur in executing those key steps, and potential solutions for these challenges. We then evaluated the relative importance of these challenges and solutions in eSource site start-up.

## MATERIALS AND METHODS

### Design

We conducted a qualitative integrative analysis with three components (16). First, we assembled participants with experience in eSource enabled clinical trials. Second, we conducted workshops to identify eSource site start-up key steps, challenges in executing those steps, and potential solutions to those challenges. Third, we conducted a value analysis to determine the challenges and solutions with the greatest impacts for eSource implementation teams. This study’s protocol (20230753X) was reviewed by the University of Texas Health Sciences Center at San Antonio Institutional Review Board and the study was determined to be exempt.

### Subjects and Setting

Workshop participants were identified from the Society for Clinical Data Management (SCDM) eSource Implementation Consortium’s membership. Our objective was to include representation from study sponsors and study sites. All candidates had direct experience with secondary use of electronic health record (EHR) data for clinical research or were Governing Members of the SCDM eSource Implementation Consortium. Study management also included an eSource vendor with an FDA-funded eSource project. Candidates were invited to participate via email.

### Evaluation Analysis

Workshop participants identified: (1) Sponsor and Site Key Steps that could be impacted by each Challenge and (2) Challenges that could be mitigated by each Solution. This information was then used to calculate two study measurements.

- Challenge Importance: This measurement was calculated as the number of Sponsor or Site Key Steps potentially impacted per Challenge.
- Solution Importance: This measurement was calculated as the number Challenges potentially mitigated per Solution.

## RESULTS

### Study Population

The first workshop was held on May 25, 2023, the second on July 26, 2023 and the third on September 18, 2023. Participants included 10 pharmaceutical sponsor, 5 academic site, and 1 eSource vendor representative (Table 1). Both groups had significant relevant experience. This included clinical data management (sponsors, mean 16.9 years, standard deviation 10.0 years; sites and vendor mean 19.0 years, standard deviation 8.5 years), clinical research (sponsors, mean 15.5 years, standard deviation, 9.5 years; sites and vendor mean 21.4 years, standard deviation 7.2 years), and eSource experience (sponsors, mean 6.7 years, standard deviation, 5.2 years; sites and vendor mean 7.1 years, standard deviation, 5.2 years). During the workshops, participants identified Site Start-up Key Steps for sponsors and sites, Challenges associated with enacting the Key Steps, and potential Solutions for the Challenges. Both groups contributed to this work (sponsors, mean 2.3 sessions per participant, standard deviation, 0.7 sessions; sites and vendor, mean 2.3 sessions per participant, standard deviation, 0.8 sessions).

### Site Start-Up Key Steps

Participants identified 36 Site Start-Up Key Steps for eSource enabled studies, 19 for sponsors and 17 for sites (Table 2). The Key Steps were divided into three groups denoting associations with: (1) Site Feasibility / Site Selection, (2) Contracting, and (3) Setting-Up. Site Feasibility / Site Selection included 17 key steps, 10 for sponsors and 7 for sites. These activities seek to determine whether a site is able to participate as an eSource site in a sponsored study. eSource Contracting included 8 key steps, 3 for sponsors and 5 for sites. These activities address the legal and regulatory aspects of sponsor-site eSource studies. Setting-Up included 11 key steps, 6 for sponsors and 5 for sites. These activities address the technical activities associated with a site’s participation in a specific eSource study.

### Site Start-Up Challenges

Participants identified 11 Site Start-Up Challenges for eSource enabled studies: 6 Challenges were associated with Site Feasibility/Site Selection Key Steps, 2 Challenges were associated with Contracting Key Steps, and 3 Challenges were associated with Setting-Up Key Steps (Table 3). Participants identified 77 potential impacts of these Challenges upon the Site Start-Up Key Steps (44 potential impacts for Sponsor and 33 for Site Key Steps) (Table 4). Most potential impacts of Challenges upon Site Start-Up Key Steps were related to Site Feasibility / Site Selection (58 total, 37 for Sponsor and 21 for Sites). There were only 8 Contracting Challenges identified (3 for Sponsor and 5 for Sites) and 11 Setting-Up Challenges identified (4 for Sponsor and 7 for Sites) with potential impacts on Site Start-Up Key Steps. The Site Feasibility / Site Selection Challenges most impacting Site Start-Up Key Steps were: Not able to identify a site champion (77% Total Key Steps impacted, 80% for Sponsors and 71% for Sites) and No decision on eSource approach (77% Total Key Steps impacted, 90% for Sponsors and 57% for Sites). The Contracting Challenge most impacting Site Start-Up Key Steps was: eSource contracting requires a new type of contract between sponsors and sites (75% Total Key Steps impacted, 67% for Sponsors and 80% for Sites). The Setting-Up Challenge most impacting Sponsor Key Steps was: Creating a site data dictionary, and agreement on what data will be included is an iterative, time-consuming process (64% Total Key Steps impacted, 50% for Sponsors and 80% for sites).

### Site Start-Up Solutions

Participants identified 14 Site Start-Up Solutions for the eSource Site Start-Up Challenges: 3 Solutions were associated with Site Readiness, 4 Solutions were associated with Contract Readiness, and 7 Solutions were associated with Technical Readiness (Table 5). Participants identified 70 ways Site Start-Up Solutions might impact Site Start-Up Challenges (45 for Site Feasibility / Site Selection, 12 for Contracting, and 13 for Setting-Up) (Table 6). These potential impacts were distributed somewhat evenly across Solution areas (19 for Site Readiness, 23 for Contract Readiness, and 28 for Technical Readiness).

The Site Readiness Solutions most impacting Site Start-Up Challenges was: Create common site self-assessment readiness checklist (64% Total Challenges impacted, 100% for Site Feasibility / Site Selection, 50% for Contracting, and 0% for Setting-Up). The Site Contracting Solutions most impacting Site Start-Up Challenges were: Create standard content for all eSource-related legal documents (73% Total Challenges impacted, 67% for Site Feasibility / Site Selection, 100% for Contracting, and 67% for Setting-Up) and Create data transfer specifications/types and mappings that enable scaling to be added to scope of work contract language (64% Total Challenges impacted, 83% Site Feasibility / Site Readiness, 0% Contracting, and 67% Setting-Up). This information can then be added to contract language regarding Scope of Work for sites and vendors. The Technical Readiness Solution most impacting Site Start-Up Challenges was: Recommend that sponsor vendors accept electronic data in the FHIR standard (82% Total Challenges impacted, 83% for Site Feasibility / Site Readiness, 0% for Contracting, and 67% for Setting-Up).

## DISCUSSION

Workshop participants identified 36 Site Start-Up Key Steps for eSource-enabled studies, 11 Site Start-Up Challenges to completing those Key Steps, and 14 potential Solutions for mitigating the Challenges. Challenges were rated according to their impacts (number of Key Steps impacted) and Solutions were rated according to their impacts (number of Challenges potentially impacted). While the implementation and use of eSource software is often presented as a technical problem, this study’s participants saw eSource implementation as a complex socio-technical problem that requires cooperation between sponsor and sites information technology and clinical research personnel.

Historically, the use of new information technologies has required the re-engineering of key workflow processes and the creation of designs that improve usability (17, 18). However, a recent study found that most clinical trial sites are not prepared to use eSource technologies in multi-center clinical studies (9). This lack of preparation is most evident in lack of meaningful interaction between the site’s information technology group and site-based clinical research teams. Many of the better prepared sites had re-engineered their information technology groups and processes to create separate groups to support clinical research. The present study contributes to this dialogue by proposing solutions that potentially will re-engineer site support for eSource enabled studies.

Implementing eSource capabilities in multi-center clinical trials may be challenging due to the time constraints faced by sponsor and site clinical research teams. To be successful, eSource data collection must integrate with existing site workflows. Fortunately, many of this study’s proposed Site Start-Up Solutions can be addressed before a specific clinical trial is identified. Thus, it is feasible to use a common site self-assessment readiness checklist to evaluate prospective eSource sites. Similarly, eSource-related legal documents with common content can be used and data mappings can begin before a specific study is identified. These documents should be aligned on site-sponsor data and workflow scenarios and their definitions should enable strategic discussion with a sponsor and potential sites.

This study has several limitations. First, the proposed Site Start-Up Solutions have not been tested and evaluated in multi-center clinical trials. Nevertheless, this study’s participants have extensive experience with eSource studies and the solutions represent their best attempts at mitigating known Challenges. Second, while sites may want data exchanged via FHIR, many sponsors and EDC vendors are not ready for this approach. Third, it is possible that new study types (e.g., decentralized, real world evidence, and registry-based clinical trials) may create new challenges and opportunities for eSource-enabled studies. Nonetheless, we believe that by addressing Solutions for known Challenges we have started a process that will lead to improvements in eSource site start-up.

## CONCLUSIONS

Site start-up for eSource-enabled multi-center clinical trials is a complex socio-technical problem. The Start-Up Challenges and Solutions identified by this study’s participants provide a basis for future work that will create an infrastructure for eSource implementation that is scalable.

## Figures and Tables

**Table 1:** Workshop Participants

Name	Organization	Role	Perspective
Mike Buckley	Memorial Sloan Kettering Cancer Center	Associate Director, Product Management, Clinical Research Informatics and Technology	Site
Peter Casteleyn	Johnson & Johnson	Director Clinical Data Collection Solutions - EHR	Sponsor
Cal Collins	OpenClinica	Chief Executive Officer	eSource Vendor
Amy Cramer	Johnson & Johnson	Director Innovative Health Innovation	Sponsor
Hugh Dai	Eli Lilly	Associate Director of Clinical Trial Design Capabilities	Sponsor
Eric Eisenstein	Duke University	Associate Professor Emeritus in Medicine	Site
Cory Ennis	Duke University	Asst. Dean-Research Systems, Strategy & Innovation	Site
Muayad Hamidi	University of Texas Health Science Center San Antonio	Clinical Research Informatics Specialist	Site
Linda King	Astellas Pharma	Director of Global eCOA/eSource Capability, Data Management, Data Science	Sponsor
Muzafar Mizra	Pfizer	Senior Group Lead, Clinical Data Sciences, Oncology (Hematology)	Sponsor
Claudine Moore	AbbVie	Program Lead, Oncology, Clinical Data Strategy and Operations	Sponsor
John Rice	Johnson & Johnson	Communications Consultant/Contractor for J&J	Sponsor
Goncalo Rodrigues	Johnson & Johnson	EMEA Medical Program Lead	Sponsor
Denise Snyder	Duke University	Associate Dean for Clinical Research	Site
Aruna Vattikola	Merck & Co., Inc.	Director, eClinical Capabilities Lead, eClinical Technologies, Global Data Management & Standards	Sponsor
Mike Ward	Eli Lilly	Senior Director of eSource Capabilities	Sponsor

**Table 2:** Site Start-Up Key Steps

Key Steps	
Title	Descriptions

**Site Feasibility / Site Selection**	
** Sponsor Steps**	
A. Site selection criteria	Sponsors develop eSource site selection criteria. Typically, these include factors associated with the site’s: (a) interest in eSource study participation (e.g., leadership engagement), (b) technical compliance and (c) potential eSource data volume (assessed by data availability, number of studies conducted, number of patients enrolled).
B. Potential technology, vendors/partners	eSource vendor selection will consider whether the site’s preferences meet the sponsor’s compliance and data privacy requirements. Geographical differences in regulations and the need for language translation may influence vendor selection.
C. Site engagement / Assess site’s capabilities	Site capabilities assessments are focused on: (a) the use of an EHR with data that is discrete and complete, (b) data standards utilization (e.g., LOINC, SNOMED, RxNORM), and (c) the technological experience of implementation team.
D. Site Technology Discussions (% of paper vs. EHR)	Site technology discussions involve site and sponsor representatives. Depending upon the site’s preference, vendors may be included. These discussions typically focus on a specific study at a specific site as scaling is site by site
E. Scalability to other sites, for the same trial.	An eSource strategy must be scalable and meet compliance, regulatory, and privacy requirements to be viable.
F. Develop site champions to accelerate implementation	There is no clear process for sponsors connecting with a site’s research IT group. Typically, the sponsor or a vendor contacts the chief research information officer (CRIO) or equivalent.
G. Protocol, MOP, abstraction guidelines, training plan	Sponsor playbooks will recognize eSource use in the study protocol, MOP, abstraction guidelines, and training plan. These may include vendor-developed training materials. Sites may create their own documentation and training materials for their study team.
H. Budget	Sponsor eSource budgets typically include funding for internal resources and a small stipend for sites. There may be a separate budget for the eSource project.
I. Recruitment plan	Creating a site eSource recruitment plan is challenging because of the dependencies with the site’s clinical research interests. Most sponsors have sites that have requested eSource or are high on the selection criteria checklist. These can provide a starting point for site recruitment.
J. High level process flow (DM Plan SDV approach)	These processes are sponsor dependent. Many sponsors will use SDV/SDR when the eSource data flow is initiated and then periodically reduce SDV to zero while retaining SDR. However, this approach is dependent upon a vendor’s ability to identify and triage data transfer issues.
** Site Steps**	
A. Identify internal stakeholders/ implementers / approvers	Typically, the CRIO or equivalent will work with the site study team and the sponsor to identify internal stakeholder, implementers, and approvers.
B. Determine if site/sponsor architecture aligns	Does site’s eSource fit with the sponsor’s eSource options?
C. Ensure site satisfies all site selection criteria	The CRIO or equivalent will work with the site study team and the sponsor to ensure the site selection criteria are satisfied
D. Identify and ensure fitness of potential technology vendors/parties	During the software development lifecycle (SDLC) process, the sponsor will work with the CRIO or equivalent to identify and ensure fitness of potential technology vendors/parties.
E. Finding a champion / study staff aligned / see potential benefit	At the time of configuration, the sponsor will work with the CRIO or equivalent to identify a champion with an ongoing study for integration testing.
F. Study selection (~6 months)-	It is important to select a study with sufficient data to exchange to obtain a favorable ROI. Factors to consider include the likely patient sample size and data types being exchanged.
G. Ensure that necessary resources are available	CRIO or equivalent and site study team work with sponsor to ensure necessary resources are available to study team.
**Contracting**	
** Sponsor Steps**	
A. Application appropriately documented	This step relates to the sponsor’s internal eSource documentation needs (e.g., across regions regulatory compliance analysis, security and privacy risk assessment, and master agreement).
B. Application available for installation	eSource vendor assures sponsor and site CRIO or equivalent that application is available for installation.
C. Agreements executed	Three types of agreement are needed: site to vendor, site to sponsor, vendor to sponsor agreements. Typically, a site to vendor agreement is something like a BAA, a site to sponsor agreement preferably consist of a master clinical trial agreement and potentially a service agreement for the first setup, and a vendor to sponsor agreement would comprise of a master agreement and a separate workorder for site implementation.
** Site Steps**	
A. Budget	There are two types of eSource site budget: (1) a budget to enable the eSource data pipeline and (2) a budget for data pipeline maintenance.
B. Funding approach	Sites may rely upon sponsor budget for setup costs. Challenges arise when sponsors fund site integration with yearly project budgets that may not correspond to a study’s duration.
C. Security and compliance sign-off	Security and compliance sign-off applies to sponsors, vendors and sites. For the sponsor, the eSource team creates the documents, presents all required legal, compliance, risk, procurement, and privacy documents for sign-off and then final approval by project sponsors. Vendors supply this information for sites to use. Site will assure the security of the 3^rd^ party middleware/vendor via their audits and testing.
D. Contracts language	Contract language needs to be aligned between vendors, sites, and the sponsor. One solution is to begin with a master Clinical Trial Agreement and use Work Order addenda language for study specific considerations.
E. Required agreements (MSA/CDA, etc.)	Sponsors typically initiate these types of agreement with red line reviews by sites.
**Setting-up**	
** Sponsor Steps**	

A. Determine data element specifications	Data element specifications typically are agreed upon by the site, vendor and the sponsor based on site preferences, USCDI, the repeatability of data elements, and vendor limitations.
B. All vendor system mappings to study database	Mappings are completed by the site, vendor and sponsor. Mock data is exchanged to confirm 100% data mapping.
C. Define test approach	eSource testing is completed before user acceptance testing.
D. Site system validation	Site data source is validated as being regulatory compliant.
E. Determine where data will land	All data transferred from site systems should match specific trial requirements.
F. Validation of E2E integration	Site, sponsors and vendors validate that site data is accurately transformed by the middleware and documented in the EDC system.

**Site Steps**	
A. Site-sponsor dictionary	Optimally, the site will establish a library of core element data mappings common to all trials. Study specific mappings can be added to the library as needed.
B. Alignment on data scope/elements	eSource data scope is determined by site mapped dictionary.
C. Technical setup	Technical setup seeks to facilitates data transfer, flow, and integration. It includes data transfer specifications, dataflow diagrams, validation/testing of the integration, establishing user accounts, and any API setup.
D. Validating the integration	Sites and sponsors validate the integration using deidentified data.
E. Study team training	Sponsors and vendor conduct study team eSource training that is study and site specific. Site personnel involved include site principal investigators (PI), clinical research associate (CRA), and monitors.

**Table 3:** eSource Site Start-Up Challenges

**Site Feasibility / Site Selection**
1. Not able to identify a site champion
2. No decision on eSource approach
3. Issues with site infrastructure, equipment, workforce, space, and readiness
4. Misalignment with eSource vendor capabilities
5. Site EHR data needed for protocol is limited not justifying ROI
6. Systems not compatible for mapping from EHR to middleware and EDC

**Contracting**
1. eSource contracting requires a new type of contact between sponsors and sites
2. IRB and regulatory compliance issues

**Setting-Up**
1. Staffing turnover prior to start up completed
2. Creating a site data dictionary, and agreement on what data will be included is an iterative, time-consuming process
3. Location of stored mappings may be problematic (vendor or site)

**Table 4:** Site Start-Up Challenges and Key Step Relationships

	Site Start-Up Key Steps Impacted		
Challenges	Sponsor	Site	Total

Site Feasibility / Site Selection	N=10	N=7	N=17
1. Not able to identify a site champion	8(80)	5(71)	13(77)
2. No decision on eSource approach	9(90)	4(57)	13(77)
3. Issues with site infrastructure, equipment, manpower, space, and readiness	6(60)	4(57)	10(59)
4. Misalignment with eSource vendor capabilities	6(60)	4(57)	10(59)
5. Site EHR data not sufficient to justify ROI for protocol	3(30)	2(29)	5(29)
6. Systems not compatible for mapping from EHR to middleware and EDC	5(50)	2(29)	7(41)
Total Group Key Step Impacts	37	21	58

	Sponsor	Site	Total
Contracting	N=3	N=5	N=8
1. eSource contracting requires a new type of contact between sponsors and sites	2(67)	4(80)	6(75)
2. IRB and regulatory compliance issues	1(33)	1(20)	2(25)
Total Group Key Step Impacts	3	5	8

	Sponsor	Site	Total
Setting-Up	N=6	N=5	N=11
1. Staffing turnover before start up completed	0(0)	1(20)	1(9)
2. Creating a site data dictionary, and agreement on what data will be included is an iterative, time-consuming process	3(50)	4(80)	7(64)
3. Where mappings will be stored may be problematic (vendor or site)	1(17)	2(40)	3(27)
Total Group Key Step Impacts	4	7	11
Total All Key Step Impacts	44	33	77

Note: cell values are n(%)

**Table 5:** eSource Site Start-Up Solutions

**Site Readiness**
1. Create common site self-assessment readiness checklist
2. Describe possible scenarios for site and sponsors eSource implementation
3. List site roles required for approving and implementing integration capabilities

**Contract Readiness**
1. Create standard content for all eSource-related legal documents (CDA, study agreement, site eSource data collection, and sponsor contracts)
2. Develop a master agreement with standard costing
3. Create data transfer specs/types and mappings that enable scaling to be added to scope of work contract language for sites and vendors
4. Develop a common model for contracting between the three partners: sites, data brokers, and sponsors

**Technical Readiness**
1. Develop a framework for sharing center data
2. Create mapping tools for FHIR to EDC and USDM
3. Regulatory Considerations on Site Qualifications
4. Provide guidance on Business Continuity Plan from site perspective - could be covered under the MSA
5. Develop common data transfer specs, and recommend transfer frequency based upon study type/phase.
6. Identify sponsor common core data elements and cross reference with site EHR discrete data elements
7. Recommend that sponsor vendors accept electronic data in the FHIR standard

**Table 6:** Site Start-Up Solution and Challenge Relationships

	Site Start-Up Challenge Areas			
Solutions	Site Feasibility / Site Selection	Contracting	Setting-Up	Total
Site Readiness	N=6	N=2	N=3	N=11
1. Create common site self-assessment readiness checklist	6(100)	1(50)	0(0)	7(64)
2. Describe possible scenarios for site and sponsors eSource implementation	5(83)	1(50)	0(0)	6(55)
3. List site roles required for approving and implementing integration capabilities	4(67)	2(100)	0(0)	6(55)
Total Challenge Area (n)	15	4	0	19

Contract Readiness				
1. Create standard content for all eSource-related legal documents (CDA, study agreement, site eSource data collection, and sponsor contracts)	4(67)	2(100)	2(67)	8(73)
2. Develop a master agreement with standard costing	2(33)	1(50)	1(33)	4(36)
3. Create data transfer specs/types and mappings that enable scaling for incorporation into Scope of Work agreements with sites.	5(83)	0(0)	2(67)	7(64)
4. Develop a common model for contracting between the three partners: sites, data brokers, and sponsors	2(33)	1(50)	1(33)	4(36)
Total Challenge Area (n)	13	4	6	23

Technical Readiness				
1. Develop a framework for sharing center data	3(50)	2(100)	1(33)	5(45)
2. Create mapping tools for FHIR to EDC and USDM	2(33)	0(0)	1(33)	4(36)
3. Regulatory Considerations on Site Qualifications	1(17)	1(50)	0(0)	1(9)
4. Provide guidance on Business Continuity Plan from site perspective - could be covered under the MSA	0(0)	1(50)	1(33)	2(18)
5. Develop common data transfer specs, and recommend transfer frequency based upon study type/phase.	2(33)	0(0)	1(33)	4(36)
6. Identify sponsor common core data elements and cross reference with site EHR discrete data elements	4(67)	0(0)	1(33)	6(54)
7. Recommend that sponsor vendors accept electronic data in the FHIR standard	5(83)	0(0)	2(67)	9(82)
Total Challenge Area	17	4	7	28
Total All Challenge Areas	45	12	13	70

Note: cell values are n(%)
